# 

**Published:** 1875-12

**Authors:** 


### INDEX

TO

### HALL'S JOURNAL OF HEALTH,

### FOR 1875.

A Trying Time........ 56
Active Oxygen........ 69
A Reform Needed........103
A Very Important Question........128
A Remedy for Vomiting........136
A Dangerous Practice........138
An Omission........145
A Vegetable Diet........165
Adhesive Plaster........167
A Better Way........174
A Fortune for Barbers........186
An Act of Humanity........193
A Wrong Righted........231
A Great Book........247
A Broken Heart........249
A Gentleman in Disguise........250
Amateur Printing........260
Anatomy in Schools........261
A Drugged Swimmer........269
A Remedial Institution........272
Alcohol and Strychnine........292
Artificial Teeth........293
A Blessing or a Curse........297
A Growing Vice........298
A Word to Wives........300
A Word for Women........312
A Revolution........319
A Great Industry........320
A Saving Salt........323
A Thing of Beauty........326
A Genuine Convenience........327
A Good Woman's Thoughts........362
A Word for Woman........363
A Great Achievement........369
A Deadly Poison........376
Advertised Medicines........378
A Plan for Getting Work........392
Alcohol and Longevity........397
A Sleepless Guardian........403
Analysis of Ale........404
A Lucky Man........407
A Vulnerable Point........408
Borrowing Trouble........172
Brain-Disease........130
Bad Breath........194
Bilious........259
Bleeders........285
Bad Feet........388
Caution........ 35
Condemned Food........ 45
Convalescence........ 52
Cosmetics........ 54
Clean Teeth........121
Cheese as Food........123
Cholera Infantum........151
Coffee vs. Gout........167
Cooks that Kill........173
Chronic Diseases........389
Care of the Eyes........393
Clean Cellars........177
Cremation........188
Calla Lilies........190
Care of the Eyes........237
Cutaneous Eruptions........205
Cotton Felt........256
Comeliness and Comfort........322
Choice Wheat Product........303
Curable Diseases........350
Caution........356
City of Hygeia........399
Cast-Iron Heating Devices........409
Chapped Hands........411
Dangers of Travel........ 21
Distortions of the Feet........ 24
Diphtheria........ 37
Disposing of the Dead........ 58
Deformed Feet........95, 133
Decaying Teeth........ 96
Dine Early and Eat Less........134
Don't Take Down the Stove........158
Don't Try to do Too Many Things........176
Diseases of Summer-Time........235
Diseased Meat........199
Dress Reform........263
Dinner or Lunch........291
Drain and Air the Land........294
Darning by Machinery........305
Drs. Hall and Gibbs........312
Dry Cold for Piles........328
Encourage the Boys........ 62
Eating Wood........ 94
Ear-Ache........278
Electricity and Life........119, 212
Electricity: Its Uses and Abuses........295
Feeble Females........ 46
Female Education........ 55
Furnace Heat........ 63
Feeding the Sick........ 72
Fever and Ague........153
Flowers........168
Fuchsin as an Antiseptic........253
For Services Rendered........260
Food for Teething Children........289
Fine Flour Bread........245
Give us Long Life........ 19
Genuine Charity........120
Good-Natured People........170
Granulated Wheat Biscuits........227, 288, 350
Graham Bread........238
Good Breeding........299
Good Way to Cook Apples........411
Health of Great Cities........ 8
House-Building Materials........ 15
Huddling Human Beings........ 61
How Bones Heal........100
Heliotype........125
Home Adornment........196
Hash........147
How Tobacco Hurts........275
Health and Religion........211
Heart Disease: Its Causes and Treatment........225
Household Gods........234
Hearty Breakfasts........245
Habits of Cleanliness........280
Health Food Company........281, 290, 401
How Boys Make Money........283
Healthy Eating........284
High Pressure........290
How Shall we Warm our Houses........296
Habit........300
Health and Exercise........316
Health and Comfort........325
How to Prevent Colds........329
How to Sweep a Room........366
Hygiene for the Aged........372
Infant Mortality........ 28
Intemperance........ 32
Inhaling Bad Air........ 39
Indigestible Substances........ 48
Inquiries Answered........ 88
Immortality of the Eye........ 93
Inhalation........154

### INDEX.

Infantile Diseases....193
Insanity in Horses....221
Innoculation from Soap....279
Indian Superstition....286
Is Cancer Curable....377
Keep your feet warm....40.400
Killing by Contraction....390
Let nothing be wasted.... 17
Libraries.... 36
Lime in Food....146
Longevity....171
Long Dresses....299
Lectures to Ladies....365
Lifting for Health....415
Milk Diet.... 9
More Light Sought.... 47
Malignant Throat Diseases.... 67
Modes of Dying.... 75
Mrs. Lyman's Letter.... 86
Milk Granules....129
Making an Invalid....149
Mistakes Matrimonial....167
Mothers and Nurses....169
Mental and Physical Suffering....197
Measles and Scarlet-Fever....202
Medical Electricity....219
Mightier than the Pen....324
Meat and Poison....344
Man and Other Animals....391
Making a Home....405
Nature's Uniformity.... 7
Nervous Diseases....133
New Cure for Piles....237
Object of Eating.... 6
Opium and Religion.... 7
Our Home—Dansville....26, 235
Our Summer at Maplewood....41, 77, 105, 139, 181, 205, 239, 273, 301, 333, 379
Ornament and Use.... 65
Our Dumb Animals....127
Our Battle and Our Foes....159
Overworked Schoolgirls....179
Oatmeal....180
One Woman's Work....354
Our Own....368
Painting the Lily.... 16
Purifying the Skin.... 60
Pneumonia and Steam-Coils.... 89
Pauperism and Crime....104
Personal and Organized Charity....116
Privies and Vaults....118, 286
Poisoning by Matches....204
Poisoning by Plants....257
Piles or Hemorrhoids....282
Pomarius....289, 296
Protecting the Lungs....311
Palate and Stomach....314
Packer's Products....355
Poisoning Pickles....357
Rheumatism.... 20
Removal.... 37
Richmond Hill.... 84
Rich Without Money....169
Rheumatism and Dyspepsia....204
Restaurants....248
Remarkable Gloves....358
Respect the Burden....367
Reason and Insanity....398
Substances in the Nose.... 12
Sanitary Science.... 14
Supplying New Blood.... 18
Stone or Gravel.... 21
Sleeplessness.... 44
Strange if True.... 50
Scientific Investigations.... 53
Spring Medicine.... 91
Sweetening the Food of Animals....124
Self-Inflicted....137
Spider Cancer....150
Strength....155
Shall we Possess our Soles in Comfort....161
Self-Remedied....215
Sideboard Refrigerator....236
Summer Complaint of Children....236
Save the Forests....246
Soap which Heals....255
Suicides....287
Starvation vs. the "Ruling Passion"....298
Saratoga in Winter....311
Secret of Good Looks....335
Solid Leaven....318
School Outrages....331
Sympathy for the Erring....332
Sickness Prevented....359
Still Water....364
Science in Story....365
Sewer-Gas....394
Soothing and Healing....396
Sometime....416
The Sunday Question.... 3
To Filter Water.... 10
Take Time by the Forelock.... 13
Tenement Houses.... 20
The House of Steinway....23, 64, 87, 117, 157, 189
The Teeth....33, 210, 349
Twenty-Second Year.... 34
The Fool-Killer.... 49
The Vice of Parsimony.... 61
The Best Dentifrice.... 65
The Economic.... 66
The Air we Breathe....68, 331
The Perfect Man.... 73
The Most Deadly Disease.... 83
Tea....113
The Guillotine....122
The "Utility"....132
The Contagion of Scarlet-Fever....144
Thermometers....146
The Chief Adornment....156
The Toil of Shop-Girls....169
Tobacco....172
Things....176
Trichiniasis....191
Tooth-Tablets....192
The Dry-Earth System....212
Tar in Skin-Diseases....218
The Art Preservative....220
Tyranny Over Women....237
Texas Beef....248
The Mystery of Dreams....265
To Save the Drowning....270
The Hours of Sleep....313
The Waves Defied....321
The Marvellous Gum....328
Thanksgiving-Day....347
The Flour of the Family....352
The Business Outlook....353
True Temperance....356
To Invalids....358
Two Thanksgivings....359
The Ballot for Women....360
The American Popular....360
Taming the Appetite....375
Use of Silence....168
Unfermented Wine....188
Vinegar and Flesh.... 90
Visiting the Sick....114
Ventilating Sewers....178
Whiskey-Drinking.... 11
Warming Houses.... 31
Woman's Apparel....101
Water in Pain....131
Women as Barbers....187
Words to the Young....200
Where Shall We Spend the Summer....222, 251
Wearing Flannel....330
Why We Die....332
Weak Food....343
Worthless Drugs....348
Woman's Temperance Convention....355
Whole Wheat Flour....376
What Shall We Drink....410
Young Women....171
Young Men's Christian Association....352
Zero Market-Basket....258
Zero Refrigerator....297

